# A fresh look at thiamine deficiency—new analyses by the global thiamine alliance

**DOI:** 10.1111/nyas.14594

**Published:** 2021-05-03

**Authors:** Megan W. Bourassa, Gilles Bergeron, Kenneth H. Brown

**Affiliations:** ^1^ Nutrition Science The New York Academy of Sciences New York New York; ^2^ Department of Nutrition University of California Davis Davis California

**Keywords:** thiamine, thiamine deficiency disorders, beriberi, vitamin B_1_

## Abstract

Severe thiamine (vitamin B_1_) deficiency is generally regarded as a problem affecting mostly infants in low‐income communities of Southeast Asia and adult alcoholics regardless of their location. However, recent scholarship shows that the disorders associated with thiamine deficiency may also affect heretofore unsuspected populations, and that the scope of disorders, including some long‐lasting neurocognitive consequences, is broader than previously thought.

## Introduction

In 2017, the New York Academy of Sciences, with funding from the Bill & Melinda Gates Foundation, assembled a task force to explore the global prevalence and disease burden from thiamine deficiency, and achieve consensus on critical gaps in knowledge. The task force confirmed that beriberi and other manifestations associated with thiamine deficiency likely remain a major cause of disease in many parts of the world but that data to estimate its global prevalence are insufficient.^1^ Factors that hamper accurate estimations include the paucity of laboratory resources to carry out assessments, lingering uncertainty about which biomarkers to use, and lack of consensus on cutoffs that define deficiency, as well as the lack of clear case definitions to positively diagnose instances of thiamine deficiency. At the prevention and control level, the task force also pointed at the need to better organize the public health response through supplementation, fortification, and dietary changes. Moreover, health professionals, both nationally and globally, need increased information and training in detecting, tracking, and treating thiamine‐related ailments.

The task force report motivated a global stakeholder meeting in Luang Prabang in November 2019 that brought together scientists, local health officers, and program implementers concerned with the issue. The meeting was instrumental in furthering our understanding of the global footprint of the disease, identifying populations at risk and identifying promising directions in diagnosing thiamine deficiency and treating its manifestations. A recent special issue of *Annals of the New York Academy of Sciences* ^2^ presents advances made in addressing these various aspects. The following sections briefly describe the range of material covered in the meeting and the resulting papers included in this special issue.

### Historical antecedents

The effects of thiamine deficiency were documented long before the discovery of the vitamin itself, going back to 1500‐year‐old Chinese medical texts that described the disease's telltale progressive weakness, paralysis, and high fatality rate and suggested that it was caused by “gases in the earth that enter the body through the feet, and move upward.” Subsequent texts suggest that the ailment existed throughout history in Asia and elsewhere.^3^

The dietary origin of the disease, which became known as beriberi, was not established until the 19th century when surgeons in the Dutch East Indies observed that 60% of Javanese sailors suffered from it compared with 1% of Europeans who were stationed on the same ships but consumed different foods. Similar associations with the beriberi and the diet were noted in Japan, where it was hypothesized that the low consumption of meat and high rice intake in some Asian population groups could explain the problem.^4^ Indeed, the disease virtually disappeared when local diets were modified, but the underlying cause remained obscure and was assumed to be vaguely related to protein consumption.

Some years later, Christiaan Eijkman, a microbiologist stationed in Indonesia, looked for a microbiological cause for beriberi. Unaware of previous findings, he attempted to create an animal model of the disease by exposing chickens to various bodily fluids from beriberi patients. His experiment failed to reproduce the neurological symptoms seen in humans, but he observed that chickens fed white rice were susceptible to the ailment, whereas those fed brown rice were not. Eijkman concluded that some compound present in the rice hull–the part that is removed by polishing—carried this antipolyneuritic factor.^5^ This novel idea ultimately led to the discovery of vitamins. The chemical structure of the antipolyneruitic factor was later defined in 1932 and it was officially named “thiamine.”^6^

Notwithstanding the seminal role that thiamine plays in nutrition, thiamine deficiency disorders (TDDs), thus named because of their broad array of clinical manifestations, persist to this day in much of South and Southeast Asia, and seem to be present in many other locations. The most affected population group is exclusively breastfed infants whose mothers are themselves thiamine deficient, but other demographic groups are also affected—see articles by Green *et al*., Koshy *et al*., and Gomes *et al*.^7^, ^8^, ^9^ Yet, infantile beriberi remains the most worrisome manifestation, and it accounts for up to 17% of infant mortality in Myanmar.^10^ While no other nationally representative studies have quantified the impact of TDDs on infant mortality, smaller surveys indicate that it may have a similarly high impact on infant mortality in neighboring Laos and Cambodia.^11^, ^12^

### Diagnosis

Thiamine deficiency presents many challenges to clinicians due to the broad clinical spectrum of manifestations (metabolic, neurologic, cardiovascular, respiratory, gastrointestinal, and musculoskeletal) and the frequent overlap in signs and symptoms of TDDs with other disorders, leading to misdiagnoses that can have fatal consequences or permanent neurologic sequelae. In the absence of specific diagnostic tests and recognizing the low risk of thiamine treatment, Smith *et al*. have proposed applying a low threshold of clinical suspicion and early treatment as the best approach.^13^ Building on this approach, Koshy *et al*. describe the progress made in rapidly diagnosing and treating the disorder in a secondary care hospital in Northeastern India.^8^

### Laboratory assessments

A fully vetted and reliable biomarker of thiamine status would be useful to assess the prevalence of deficiency at the population level and would be a powerful aid in the diagnosis and treatment of individual cases. Jones *et al*. present a step‐by‐step protocol for the measurement of erythrocyte transketolase (ETK) activity and calculation of the ETK activity coefficient, including a description of equipment and chemicals required, as well as guidance for quality control procedures. Harmonization of protocols is an important step toward ensuring the comparability of studies performed in different laboratory settings and over time.^14^

### Populations at risk

Thiamine deficiency has typically been associated with a reliance on poor diets in low‐ and middle‐income countries (LMICs) or with alcoholism in adults (mostly manifested as Wernicke's encephalopathy). However, as Gomes *et al*. demonstrate in their literature review, adult thiamine deficiency is not exclusive to LMICs or alcoholic patients in high‐income settings.^9^ The condition can in fact occur in patients suffering from various underlying diseases (from cancer to heart failure), due to disease‐related malnutrition, persistent vomiting, use of selected medications, such as diuretics, bariatric surgery, and other causes.

Pediatric thiamine disorders have also been observed in high‐income countries, as described in the review by Rakotoambinina *et al*., which highlighted that the predisposing factors, clinical presentations, and age distribution of pediatric thiamine deficiency differ from the patterns observed in LMICs.^15^ In high‐income settings, most cases of pediatric thiamine deficiency occur in childhood and adolescence, most commonly due to eating disorders, diabetes, obesity, excessive consumption of sweetened drinks, bariatric surgery, and cancer, and they are often manifested as hyperlactatemia and Wernicke's encephalopathy.

### Consequences of TDDs

One of the most concerning conclusions of the 2018 task force report was the potential impact of subclinical thiamine deficiency on neurocognitive outcomes for young children.^1^ An opportunistic study of Israeli infants inadvertently fed thiamine‐free formula allowed researchers to follow the impact of isolated thiamine deficiency over many years among otherwise healthy infants. Many infants who showed clinical symptoms during early infancy never fully recovered, but most worrying, a cohort of infants exposed to the thiamine‐free formula who were initially asymptomatic also showed persistent cognitive impairment in later childhood.^16^, ^17^ This led to a concern that even subclinical thiamine deficiency can have a lasting impact on developmental outcomes.^18^ To further understand the role of thiamine in cognitive development, researchers leading a placebo‐controlled, dose–response supplementation trial of lactating women in Cambodia followed the cognitive development of their breastfed infants. As described in the paper by Measelle *et al*.,^19^ infants whose mothers received the highest supplementation dose (10 mg/day) performed the highest on some cognitive assessment tests, but this advantage disappeared after supplementation ended. Interestingly, breastmilk thiamine content at 2 weeks of age, prior to the supplementation of the mothers, was highly predictive of infants’ cognitive scores up to 12 months of age.

### Control and prevention

A clear way to prevent TDDs in LMICs is to increase thiamine intake of breastfeeding mothers and others at risk of deficiency. Thiamine fortification programs have a long history in high‐income countries, but there are few mandatory fortification programs to address TDDs in LMICs. In their review, Whitfield *et al*. highlight the essential aspects to consider in developing a mandatory fortification program in LMICs, including an overview of the information required to design fortification programs, including dosing schemes, available thiamine fortificants, and potential fortification vehicles.^20^ The complex choices involved are aptly illustrated by Green *et al*. based on their experience in Kiribati.^7^ A key issue, as for any fortification program, is the choice of vehicle, itself conditioned by consumption patterns in the target location, which Chan *et al*. examine in Cambodia, where the salt intake of lactating women was previously unknown.^21^ Their rigorous estimation of salt intake in this group allowed them to establish the fortification dosage needed to reach the estimated average requirement of 1.2 mg/day for most of the population from fortified salt alone.

## Conclusion

The articles in *Ann. N.Y. Acad. Sci*., Vol. 1498 (2021),^2^ offer many advances in recognizing the severity of TDDs, their diverse manifestations, and global footprint. With greater awareness of the symptoms, biomarkers, diagnostic criteria, cognitive impacts, and treatment protocols, it is hoped that TDDs will be more accurately and rapidly diagnosed and appropriately treated or prevented to prevent unnecessary mortality and morbidity. Creating effective control and prevention programs through behavior change interventions to improve dietary diversity, food fortification initiatives, and supplementation programs will be essential to reduce the burden of thiamine‐related diseases.

## Competing interests

The authors declare no competing interests.
